# Comparisons of in-hospital complications between total hip arthroplasty and hip resurfacing arthroplasty

**DOI:** 10.1186/s12891-023-06487-7

**Published:** 2023-05-11

**Authors:** Yuanyuan Huang, Qinfeng Yang, Ziqi Wang, Zhijie Pan, Yang Zhang, Zhanjun Shi, Sheng Yang

**Affiliations:** 1 School of Health, Dongguan Polytechnic, Dongguan, Guangdong 523000 China; 2 Division of Orthopaedic Surgery, Department of Orthopaedics, Nanfang Hospital, Southern Medical University, 1838 Guangzhou Avenue, Guangzhou, Guangdong 510515 China; 3 The First School of Clinical Medicine, Southern Medical University, Guangzhou, Guangdong 510515 China

**Keywords:** Hip resurfacing arthroplasty, Total hip arthroplasty, In-hospital complications, Nationwide inpatient sample database, Osteoarthritis

## Abstract

**Background:**

Hip resurfacing arthroplasty (HRA) is a less common but effective alternative method to total hip arthroplasty (THA) for hip reconstruction. In this study, we investigated the incidences of in-hospital complications between patients who had been subjected to THA and HRA.

**Methods:**

The National Inpatient Sample data that had been recorded from 2005 to 2014 was used in this study. Based on the International Classification of Disease, Ninth Revision, Clinical Modification, patients who underwent THA or HRA were included. Data on demographics, preoperative comorbidities, length of hospital stay, total charges, and in-hospital mortality and complications were compared. Multiple logistic regression analysis was used to determine whether different surgical options are independent risk factors for postoperative complications.

**Results:**

A total of 537,506 THAs and 9,744 HRAs were obtained from the NIS database. Patients who had been subjected to HRA exhibited less preoperative comorbidity rates, shorter length of stay and extra hospital charges. Moreover, HRA was associated with more in-hospital prosthesis loosening. Notably, patients who underwent HRA were younger and presented less preoperative comorbidities but did not show lower incidences in most complications.

**Conclusions:**

The popularity of HRA gradually reduced from the year 2005 to 2014. Patients who underwent HRA were more likely to be younger, male, have less comorbidities and spend more money on medical costs. The risk of in-hospital prosthesis loosening after HRA was higher. The HRA-associated advantages with regards to most in-hospital complications were not markedly different from those of THA. In-hospital complications of HRA deserve more attention from surgeons.

## Introduction

Hip resurfacing arthroplasty (HRA) is a less common but effective alternative method to total hip arthroplasty (THA) for hip reconstruction [1, 2]. Based on its design philosophy, HRA has gained popularity among young, active patients [3–5]. However, since it was first reported in the 1940s, HRA has been associated with various complications, including femoral neck fractures, component loosening, and adverse local tissue reactions among others [2]. With continuous advances in prosthesis materials, HRA procedures have been improved, and metal-on-metal hip resurfacing, particularly Birmingham hip resurfacing became one of the most popular alternative surgeries of THA [6, 7].

Various studies have compared HRA and THA. Some studies showed that HRA improves functions and reduces pain at short- and mid-term follow-up [8, 9]. Other studies reported that HRA prosthesis has a higher revision rate than THA [10]. Besides, regarding the safety of the two procedures, THA was associated with higher mortality rates, compared to HRA [11, 12]. Overall, the main advantages of HRA were with regards to reproduction of natural joint biomechanics, conservation of femoral bone stock, meeting the functional demands and the option for future THA [6, 13]. However, the higher incidences of long-term complications after HRA have limited its clinical applications [2, 14, 15].

While the studies attempted to detect the long-term outcomes after the two procedures, a limited number of studies focused on postoperative complications during hospitalization. Therefore, we used data from a large-scale database to assess the demographics of HRA and THA patients and to detect in-hospital outcomes. We particularly focused on whether there were differences in incidences of in-hospital complications after HRA and THA.

## Methods

### Data source

The data was collected from the National Inpatient Sample (NIS) database, which is part of the Healthcare Cost and Utilization Project, Agency for Healthcare Research and Quality. The NIS represents the largest all-payer database of hospital admissions in the United States. The NIS collects a stratified sample from more than 1000 hospitals, of approximately 20% of the hospitalizations each year. Patients were included based on the diagnostic and procedural codes defined by International Classification of Diseases (ninth revision) Clinical Modification (ICD-9-CM).

### Data collection

According to the ICD-9-CM system, patients underwent THA were identified by the procedure code 81.51, and patients underwent HRA were identified by code 00.85. Exclusion criteria included pathological fractures, osteomyelitis and emergency admissions.

Patient demographics, including age, sex, and race were evaluated. Outcome measures such as length of stay, Charlson Comorbidity Index (CCI), Elixhauser Comorbidity Index, total charges, and in-hospital mortality were analyzed. Subsequently, osteoarthritis, hip dysplasia, avascular necrosis of femoral head, traumatic arthritis, fracture, rheumatoid arthritis and ankylosing spondylitis were determined as reasons for surgery and proportion of different diagnoses were calculated.

Further, perioperative complications were searched in the database based on ICD-9-CM diagnostic code. Prosthesis-related complications were defined as dislocation, periprosthetic fracture, periprosthetic joint infection, prosthesis loosening, revision arthroplasty and other prosthesis-related complications. Other perioperative complications included postoperative shock, acute posthemorrhagic anemia, blood transfusion, deep vein thrombosis, pulmonary embolism, acute cerebrovascular disease, pneumonia, acute renal failure, urinary tract infection, nerve injury of lower limb, wound dehiscence and infection, wound debridement and mortality.

### Data analysis

The statistical software, R version 3.5.3 (R Foundation for Statistical Computing, Vienna, Austria) was used to perform statistical analysis. Comparisons between the groups were performed by Wilcoxon rank sum test for continuous variables, chi-square test or fisher test for categorical variables. Multivariate logistic regression models were constructed to assess if the type of surgery influenced the postoperative complications rate independently. Odds ratios (ORs), p values, and 95% confidence intervals (CIs) of ORs were used to depict the effect instead of relying solely on statistical significance. Statistical significance was defined by p ≤ 0.001 because of the large-scale sample volume, which had been utilized by other NIS-researches.

## Results

### Trends in the number of HRA and THA

**Fig. 1:**
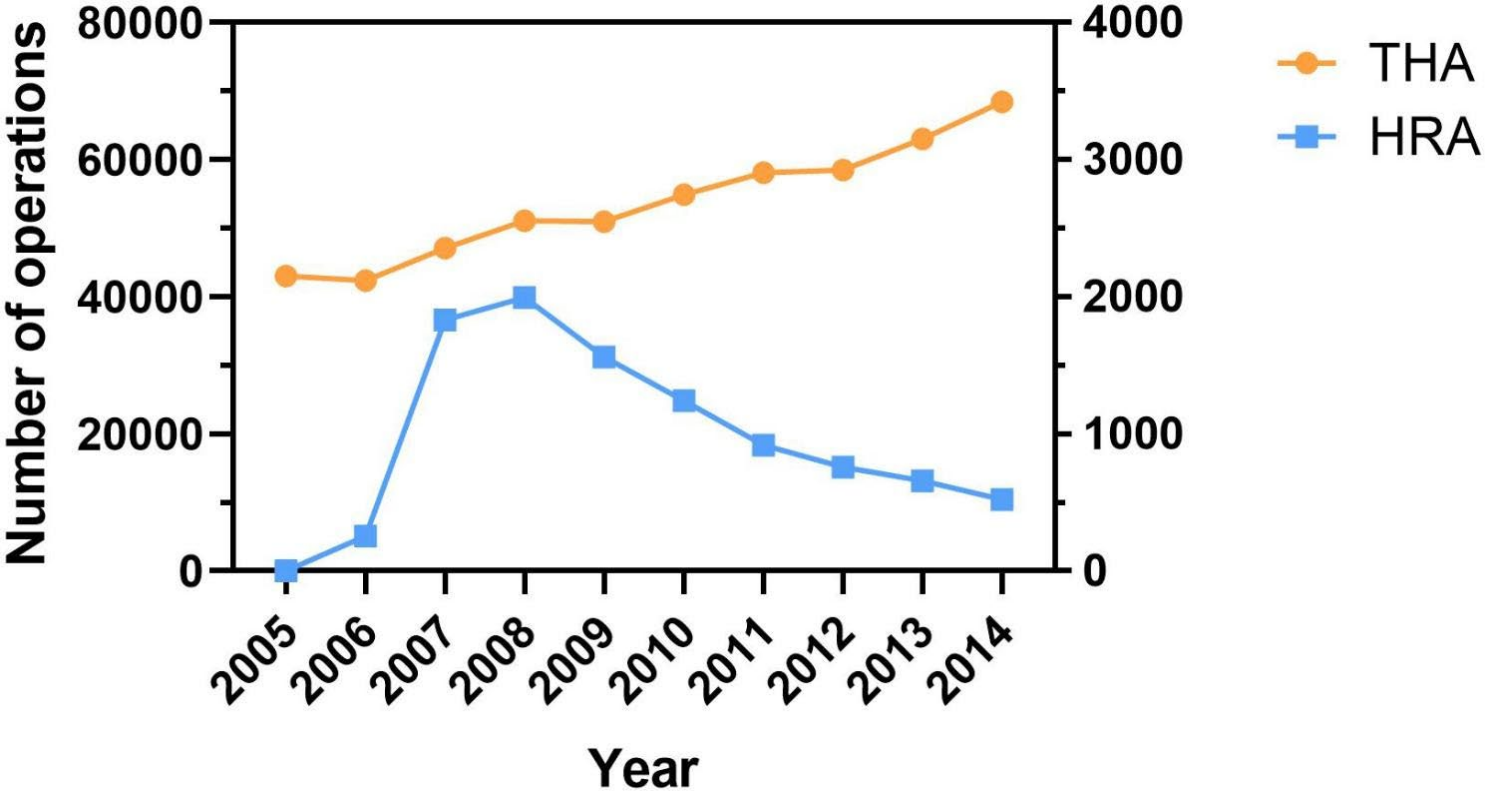
Trends in the number of HRA and THA. The left Y-axis represents the number of THA while the right Y-axis represents the number of HRA.

A total of 537,506 THAs and 9,744 HRAs were identified in the NIS database from 2005 to 2014. The number of THA cases increased annually from 2005 to 2014 (Fig. 1). More than 60,000 THAs were performed in 2014. The number of HRA cases peaked in 2008, and gradually decreased from then on.

### Demographics of patients receiving HRA and THA

Demographic data of the included cases are shown in Table 1. There were significant differences in most indicators between the groups. Patients who underwent HRA were markedly younger (52 yrs. vs. 65 yrs., p < 0.001) and less females (20.43% vs. 55.48%, p < 0.001). Differences in in-hospital mortality rates between the groups were insignificant (0.09% vs. 0.01%, p = 0.132).

**Table 1:** Demographic data of the patients who underwent Elective THA and HRA

Parameter	THA	HRA	p
Age (yrs.)	65	52	< 0.0001
Age group (%)			
0–20	0.14	0.31	< 0.0001
21–40	2.85	9.48	< 0.0001
41–60	32.59	77.94	< 0.0001
61–80	54.48	12.17	< 0.0001
≥ 81	9.94	0.09	< 0.0001
Sex (% female)	55.48	20.43	< 0.0001
Race (%)			
White	86.62	88.9	< 0.0001
Black	6.99	5.15	< 0.0001
Hispanic	3.16	2.58	< 0.0001
Asian or Pacific Islander	0.91	0.41	< 0.0001
Native American	0.31	0.17	< 0.0001
Other	2.01	2.79	< 0.0001
Charlson Comorbidity Index	3(2–4)	2(1–2)	< 0.0001
length of stay (d)	3(2–4)	3(2–3)	< 0.0001
Total charges ($)	43,802 (32,423–60,292)	47873.5 (35959.75-65693.25)	< 0.0001
In-hospital mortality (%)	0.09	0.01	0.0132
Payment type (%)			
Medicare	51.63	5.72	< 0.0001
Medicaid	3.5	1.6	< 0.0001
Private insurance	41.57	87.96	< 0.0001
Self-pay	0.73	0.91	< 0.0001
No charge	0.13	0.06	< 0.0001
Other	2.44	3.74	< 0.0001
Hospital location (% Urban)	90.42	96.23	< 0.0001
Bed size(%)			
Small	18.86	26.63	< 0.0001
Medium	24.86	20.83	< 0.0001
Large	56.28	52.54	< 0.0001

### Reasons for receiving surgeries

In Table 2, most patients in either THA or HRA groups were subjected to surgeries due to osteoarthritis. Only in less than 5% of patients was HRA not due to osteoarthritis.

**Table 2:** Diagnosis of the patients who underwent Elective THA and HRA

Diagnosis (%)	THA	HRA	p
Osteoarthritis	90.13	95.25	< 0.0001
Hip dysplasia	0.05	0.03	0.6372
Avascular necrosis of Femoral head	6.44	3.09	< 0.0001
Traumatic arthritis	0.7	0.57	0.1465
Fracture	0.54	0.01	< 0.0001
Rheumatoid arthritis	0.54	0.16	< 0.0001
Ankylosing spondylitis	0.01	0.03	0.18
Other	1.59	0.85	< 0.0001

### Preexisting comorbidities

Table 3 shows the prevalence of preexisting comorbidities. Compared with HRA, patients who underwent THA had higher incidences of preexisting comorbidities, including deficiency anemias, arrhythmia, congestive heart failure, chronic pulmonary disease, depression, uncomplicated diabetes, diabetes with chronic complications, hypertension, hypothyroidism, fluid and electrolyte disorders, other neurologic disorders, obesity, peripheral vascular disorders, psychoses, pulmonary circulation disorders, renal failure, solid tumors without metastasis, and valvular disease. Differences in incidences of acquired immunodeficiency syndrome, alcohol abuse, chronic blood loss anemia, coagulopathy, drug abuse, liver disease, lymphoma, metastatic cancer, paralysis, as well as peptic ulcer disease excluding bleeding and weight loss were not significant between the two groups.

**Table 3:** Prevalence of Comorbidities in Patients who underwent Elective THA and HRA

Comorbidities (%)	THA	HRA	p
Acquired immunodeficiency syndrome	0.14	0.08	0.1684
Alcohol abuse	1.54	1.23	0.0165
Deficiency anemias	13.49	8.38	< 0.0001
Arrhythmia	3.75	1.01	< 0.0001
Chronic blood loss anemia	1.81	2.18	0.0077
Congestive heart failure	2.37	0.19	< 0.0001
Chronic pulmonary disease	13.84	6.51	< 0.0001
Coagulopathy	2.01	2.04	0.8669
Depression	10.59	7.29	< 0.0001
Diabetes uncomplicated	13.02	4.15	< 0.0001
Diabetes with chronic complications	1.11	0.19	< 0.0001
Drug abuse	0.67	0.61	0.4825
Hypertension	58.8	30.41	< 0.0001
Hypothyroidism	12.97	5.2	< 0.0001
Liver disease	0.98	0.67	0.0021
Lymphoma	0.34	0.12	0.0004
Fluid and electrolyte disorders	8.03	3.98	< 0.0001
Metastatic cancer	0.13	0.05	0.0415
Other neurologic disorders	3.21	1.17	< 0.0001
Obesity	14.6	10.77	< 0.0001
Paralysis	0.32	0.09	0.0001
Peripheral vascular disorders	2.16	0.41	< 0.0001
Psychoses	1.71	0.92	< 0.0001
Pulmonary circulation disorders	0.7	0.18	< 0.0001
Renal failure	3.5	0.53	< 0.0001
Solid tumor without metastasis	0.51	0.14	< 0.0001
Peptic ulcer disease excluding bleeding	0.02	0	0.4295
Valvular disease	3.78	1.86	< 0.0001
Weight loss	0.38	0.13	0.0001

### Prosthesis-related complications after THA or HRA during hospitalization

Comparisons of incidences of complications and multivariate regression results are shown in Table 4. In addition to prosthesis loosening, higher incidences of most prosthesis-related complications were observed after THA, including dislocation, periprosthetic fractures, periprosthetic joint infections and revision arthroplasty. However, differences in these outcomes between the groups were insignificant. Notably, HRA exhibited a significantly high OR for prosthesis loosening (OR = 5.4054, p < 0.001). Univariate analysis revealed that incidences of ‘any prosthesis-related complications’ were markedly high in the THA group, while multivariate analysis did not yield a significant difference.

**Table 4:** In-hospital Postoperative Complications Associated with Patients who underwent Elective THA and HRA

Complication	THA	HRA	p	OR ^a^	95% CI ^b^
Prosthesis-related complications					
Dislocation	1088(0.2%)	16(0.16%)	0.47	0.97	0.38–2.53
Periprosthetic fracture	554(0.1%)	4(0.04%)	0.08	0.63	0.13–3.13
Periprosthetic joint infection	905(0.17%)	13(0.13%)	0.47	2.11	0.77–5.75
Prosthesis loosening	455(0.08%)	17(0.17%)	0.005	5.41	2.13–13.71
Revision arthroplasty	6179(1.15%)	52(0.53%)	< 0.001	1.30	0.54–3.11
Other prosthesis-related complications	1670(0.31%)	27(0.28%)	0.62	1.59	0.86–2.92
Any prosthesis-related complication ^c^	9485(1.76%)	99(1.02%)	< 0.001	0.54	0.22–1.30
Other common complications					
Postoperative shock	446(0.08%)	3(0.03%)	0.11	1.08	0.26–4.49
Acute posthemorrhagic anemia	132,010(24.56%)	1813(18.61%)	< 0.001	0.86	0.75–0.98
Blood transfusion	103,639(19.28%)	1024(10.51%)	< 0.001	0.84	0.74–1.04
Deep vein thrombosis	967(0.18%)	7(0.07%)	0.02	0.47	0.17–1.28
Pulmonary embolism	892(0.17%)	8(0.08%)	0.06	1.27	0.50–3.21
Acute cerebrovascular disease	561(0.1%)	2(0.02%)	0.016	0.96	0.23–3.98
Pneumonia	2284(0.42%)	2(0.02%)	< 0.001	0.16	0.03–0.64
Acute renal failure	8658(1.61%)	41(0.42%)	< 0.001	0.77	0.54–1.08
Urinary tract infection	15,040(2.8%)	74(0.76%)	< 0.001	0.62	0.47–0.82
Nerve injury of lower limb	237(0.04%)	11(0.11%)	0.0054	2.71	1.33–5.51
Wound dehiscence and infection	4833(0.9%)	68(0.7%)	0.042	0.97	0.72–1.31
Wound debridement	690(0.13%)	7(0.07%)	0.16	0.46	0.18–1.17
Mortality	491(0.09%)	1(0.01%)	0.01	0.45	0.06–3.63
Any common complications ^d^	199,299(37.09%)	2535(26.02%)	< 0.001	1.30	1.12–1.50

^a^ CI, Confidence Interval

^b^ OR, Odds Ratio

^c, d^ Any prosthesis-related complication or any common complications: patients with more than one complication were counted only once

### Other complications after THA/HRA during hospitalization

Detailed data are shown in Table 4. Incidences of most complications after THA were higher than after HRA except nerve injury of lower limb, of which acute posthemorrhagic anemia, blood transfusion, pneumonia, acute renal failure and urinary tract infection exhibited significant differences. Moreover, multivariable analysis did not reveal significant differences in most of the complications. Unexpectedly, the HRA group had a higher OR (OR 1.2985, p < 0.001) for ‘any common complications’ while a significant lower incidence was found in univariate analyses (26.02% vs. 37.09%, p < 0.001).

## Discussion

This study performed a large-scale analysis of in-hospital complications after THA and HRA. It is worth noting that this data represents the NIS and may not represent the surgical population as a whole.

From the year 2005 to 2014, the popularity of HRA gradually reduced. At its peak in 2008, HRA was used in 3.8% of hip arthroplasty cases. This proportion was lower compared to other studies. In England and Wales, HRA accounted for 10% of all primary total hip replacements in 2006 [16]. However, the decreasing trend is in line with reports from previous studies [17, 18]. In the past few years, several influential commentaries have even called for abolition of HRA prosthesis [19, 20]. Recent reports on resurgent use of HRA in elite sports people and the possibility to return to elite-level sporting activities have led to an increased interest from patients [21].

We found that patients who underwent HRA were significantly younger, which may be attributed to the poor long-term implant survival rate and difficulty in revision of THA [22]. In contrast, HRA is bone-preserving and therefore, potentially easy to revise [23]. There was a significant male preponderance in the HRA group. Generally, compared to female patients, male patients had larger diameters of femoral heads. A larger femoral head diameter improves the head-to-neck ratio and increases the range of motions without prosthetic impingement [24], resulting in better implant survival rates [18]. Moreover, women have an increased risk of osteoporotic fractures of the femoral neck or a greater predisposition to reactions to metal debris [25].

The importance of patient selection for HRA has been reported [3, 4, 26–28]. In this study, we found that only less than 5% of patients that underwent HRA was not due to osteoarthritis. Hip dysplasia is an independent risk factor for failure following HRA [29]. Osteonecrosis of the femoral head may result in aseptic loosening and femoral neck narrowing secondary to osteonecrosis progression after HRA [30]. Robert Sershon et al. [3] reported femoral neck fractures and aseptic loosening as the two most common long-term complications and modes of failure in FDA-approved HRA. In previous studies, incidences of short- and middle-term prosthesis-related complications between the two operations were comparable. Ran Tao et al. [31] followed 68 patients that had been subjected to HRA or THA for a period of at least 5 years. They found that differences in major complications, including loosening, fractures, dislocations, infections, and adverse reactions to the metal debris (ARMD) were insignificant between the groups. After prospective follow up for 2 to 4 years, Vincent Fowble et al. [32] compared 50 metal-metal resurfacing replacements with 44 conventional total hip arthroplasties. There were no significant differences in major postoperative complications between the treatments. However, in our study, there were higher incidences of prosthesis loosening during hospitalization after HRA (0.08% vs. 0.17%, p = 0.0048). Moreover, HRA was associated with significantly higher OR (OR = 5.4054, p < 0.001) for prosthesis loosening. Rigorous patient selection criteria for clinical studies may be one of the reasons, which led to better outcomes after HRA. The risk of prosthesis loosening after HRA should be investigated further.

Intraoperative bleeding is one of the major challenges after THA [33–35]. It has been reported that since it preserves a relatively intact femur medullary cavity, HRA can effectively reduce bleeding [36]. In this study, incidences of acute hemorrhagic anemia (24.56% vs. 18.6%, p < 0.001) and blood transfusion (19.28% vs. 10.51%, p < 0.001) after HRA were markedly lower, but lost their statistical significance in multivariate analysis. These findings suggest that bleeding outcomes after THA may be attributed to poorer basic conditions of THA patients. Similar postulates were applied to other complications. We found that HRA had higher ORs for ‘any common complications’ while it exhibited significant lower rates in univariate analyses.

Patient demographic characteristics in this study were comparable to those reported in prior studies. Nevertheless, this study suggests a higher risk of in-hospital complications, such as prosthesis loosening after HRA. Under the premise of fewer comorbidities in patients who underwent HRA, their total charges were higher. Considering the various long-term complications of HRA that have been previously reported, studies should be performed to clarify the indications of HRA.

However, a number of limitations remained associated with the present study. Firstly, the details of arthroplasty including surgery duration, head size, acetabular inclination angle and prosthesis type were not included in the NIS database. These factors influenced the final operative outcomes and could be potential confounders. Secondly, diagnosis of each patient was only recorded before hospital discharge and could not be distinguished from those diagnosis before admission. Therefore, the analysis of common complications might be imprecise, because these symptoms might already exist before surgery. Furthermore, as with any large database, there might be discrepancy or misclassification in coding and documentation. Thus, administrative data tend to have high specificity (low false positive rate) but low sensitivity (high false-negative rate) in identifying adverse events, which might also underestimate the incidence of each complication [37].

## Conclusions

In conclusion, HRA is a less common but effective alternative method to THA for hip reconstruction. The popularity of HRA gradually reduced from the year 2005 to 2014. Patients who underwent HRA were more likely to be younger, male, have less comorbidity and spent more on medical costs. The risk of in-hospital prosthesis loosening after HRA was higher. The advantages of HRA in most in-hospital complications were comparable to those of THA. On the contrary, HRA showed higher risks for some common complications. In-hospital complications of HRA deserve more attention from surgeons.

## Data Availability

This study is based on data provided by Nationwide Inpatient Sample (NIS) database, part of the Healthcare Cost and Utilization Project, Agency for Healthcare Research and Quality. The NIS database is a large publicly available all-payer inpatient care database in the United States. Therefore, individual or grouped data cannot be shared by the authors. Links to the database are as follows: https://www.hcup-us.ahrq.gov/db/nation/nis/nisdbdocumentation.jsp.

## Abbreviations

THA: Total hip arthroplasty
HRA: Hip resurfacing arthroplasty
NIS: Nationwide Inpatient Sample
ICD-9-CM: International Classification of Diseases (ninth revision) Clinical Modification
CCI: Charlson Comorbidity Index
OR: Odds ratio
CI: Confidence interval.

## References

[1] Matharu GS, Pandit HG, Murray DW, Treacy RB (2015). The future role of metal-on-metal hip resurfacing. Int Orthop.

[2] Amstutz HC, Le Duff MJ (2015). Hip resurfacing: history, current status, and future. Hip Int.

[3] Sershon R, Balkissoon R, Valle CJ (2016). Current indications for hip resurfacing arthroplasty in 2016. Curr Rev Musculoskelet Med.

[4] McMinn DJ, Daniel J, Ziaee H, Pradhan C (2011). Indications and results of hip resurfacing. Int Orthop.

[5] Girard J, Lons A, Pommepuy T, Isida R, Benad K, Putman S (2017). High-impact sport after hip resurfacing: the Ironman triathlon. Orthop Traumatol Surg Res.

[6] Daniel J, Pynsent PB, McMinn DJ (2004). Metal-on-metal resurfacing of the hip in patients under the age of 55 years with osteoarthritis. J Bone Joint Surg Br.

[7] Ford MC, Hellman MD, Kazarian GS, Clohisy JC, Nunley RM, Barrack RL (2018). Five to ten-year results of the Birmingham Hip Resurfacing Implant in the U.S.: a single Institution’s experience. J Bone Joint Surg Am.

[8] Treacy RB, McBryde CW, Pynsent PB (2005). Birmingham hip resurfacing arthroplasty. A minimum follow-up of five years. J Bone Joint Surg Br.

[9] Mont MA, Seyler TM, Marker DR, Marulanda GA, Delanois RE (2006). Use of metal-on-metal total hip resurfacing for the treatment of osteonecrosis of the femoral head. J Bone Joint Surg Am.

[10] Stoney J, Graves SE, de Steiger RN, Rainbird S, Kelly TL, Hatton A (2020). Is the survivorship of Birmingham Hip Resurfacing Better Than selected conventional hip Arthroplasties in Men younger than 65 years of age? A study from the australian Orthopaedic Association National Joint replacement Registry. Clin Orthop Relat Res.

[11] Kendal AR, Prieto-Alhambra D, Arden NK, Carr A, Judge A (2013). Mortality rates at 10 years after metal-on-metal hip resurfacing compared with total hip replacement in England: retrospective cohort analysis of hospital episode statistics. BMJ.

[12] McMinn DJ, Snell KI, Daniel J, Treacy RB, Pynsent PB, Riley RD (2012). Mortality and implant revision rates of hip arthroplasty in patients with osteoarthritis: registry based cohort study. BMJ.

[13] Suraci AB, Bhullar RS, Dobransky JS, Beaulé PE (2021). Hueter Anterior Approach for Metal-on-metal hip resurfacing arthroplasty: 555 cases at a Minimum five-year Follow-Up. J Arthroplasty.

[14] Shimmin AJ, Bare J, Back DL (2005). Complications associated with hip resurfacing arthroplasty. Orthop Clin North Am.

[15] Palazzuolo M, Antoniadis A, Delaune L, Tornare I, Wegrzyn J (2021). Comparison of the long-term cause of failure and survivorship of four hundred and twenty-seven metal-on-metal hip arthroplasties: resurfacing versus large head total hip arthroplasty. Int Orthop.

[16] Metcalfe D, Peterson N, Wilkinson JM, Perry DC (2018). Temporal trends and survivorship of total hip arthroplasty in very young patients: a study using the National Joint Registry data set. Bone Joint J.

[17] Hastie GR, Collinson SC, Aqil A, Basu S, Temperley DE, Board TN (2021). Study to assess the rate of adverse reaction to metal debris in hip resurfacing at a minimum 13-year follow-up. J Arthroplasty.

[18] Smith AJ, Dieppe P, Howard PW, Blom AW (2012). Failure rates of metal-on-metal hip resurfacings: analysis of data from the National Joint Registry for England and Wales. Lancet.

[19] Kmietowicz Z (2012). Failure rates of Birmingham hip implant exceed recommended level. BMJ.

[20] Heneghan C, Langton D, Thompson M (2012). Ongoing problems with metal-on-metal hip implants. BMJ.

[21] Morse KW, Premkumar A, Zhu A, Morgenstern R, Su EP (2021). Return to Sport after Hip Resurfacing Arthroplasty. Orthop J Sports Med.

[22] Ferguson RJ, Palmer AJ, Taylor A, Porter ML, Malchau H, Glyn-Jones S (2018). Hip replacement. Lancet.

[23] Clough EJ, Clough TM (2021). Metal on metal hip resurfacing arthroplasty: where are we now?. J Orthop.

[24] Soong M, Rubash HE, Macaulay W (2004). Dislocation after total hip arthroplasty. J Am Acad Orthop Surg.

[25] Pandit H, Glyn-Jones S, McLardy-Smith P, Gundle R, Whitwell D, Gibbons CL (2008). Pseudotumours associated with metal-on-metal hip resurfacings. J Bone Joint Surg Br.

[26] Ribas M, Cardenas C, Astarita E, Moya E, Bellotti V (2014). Hip resurfacing arthroplasty: mid-term results in 486 cases and current indication in our institution. Hip Int.

[27] Asaad A, Hart A, Khoo MM, Ilo K, Schaller G, Black JD (2015). Frequent femoral neck osteolysis with Birmingham mid-head resection resurfacing arthroplasty in young patients. Clin Orthop Relat Res.

[28] Haughom BD, Erickson BJ, Hellman MD, Jacobs JJ (2015). Do complication rates differ by gender after metal-on-metal hip resurfacing arthroplasty? A systematic review. Clin Orthop Relat Res.

[29] Gross TP, Liu F (2012). Prevalence of dysplasia as the source of worse outcome in young female patients after hip resurfacing arthroplasty. Int Orthop.

[30] Woon RP, Johnson AJ, Amstutz HC (2012). Results of Conserve Plus® metal-on-metal hip resurfacing for post-traumatic arthritis and osteonecrosis. Hip Int.

[31] Tao R, Liu F, Liu YK, Lu Y, Xu H, Cao Y (2018). A prospective comparative study of hip resurfacing arthroplasty and large-diameter head metal-on-metal total hip arthroplasty in younger patients-a minimum of five year follow-up. Int Orthop.

[32] Fowble VA, Dela RM, Schmalzried TP (2009). A comparison of total hip resurfacing and total hip arthroplasty - patients and outcomes. Bull NYU Hosp Jt Dis.

[33] Friedman RJ. Limit the bleeding, limit the pain in total hip and knee arthroplasty. Orthopedics. 2010;33 Suppl 9:11 – 3. 10.3928/01477447-20100722-62 20839716

[34] Cherian JJ, Banerjee S, Kapadia BH, Sodhi GS, Issa K, Harwin SF (2014). Nonsurgical intra-operative blood management strategies for total hip arthroplasty. Surg Technol Int.

[35] Wu XD, Xiao PC, Zhu ZL, Liu JC, Li YJ, Huang W (2019). The necessity of routine postoperative laboratory tests in enhanced recovery after surgery for primary hip and knee arthroplasty: a retrospective cohort study protocol. Med (Baltim).

[36] Shimmin A, Beaulé PE, Campbell P (2008). Metal-on-metal hip resurfacing arthroplasty. J Bone Joint Surg Am.

[37] Bozic KJ, Bashyal RK, Anthony SG, Chiu V, Shulman B, Rubash HE (2013). Is administratively coded comorbidity and complication data in total joint arthroplasty valid?. Clin Orthop Relat Res.

